# BLESeis: Low-Cost IoT Sensor for Smart Earthquake Detection and Notification

**DOI:** 10.3390/s20102963

**Published:** 2020-05-23

**Authors:** Jongbin Won, Junyoung Park, Jong-Woong Park, In-Ho Kim

**Affiliations:** 1 Department of Civil and Environmental Engineering, Chung-Ang University, Dongjak, Seoul 06974, Korea; sac1721@cau.ac.kr (J.W.); pjy5451@cau.ac.kr (J.P.); 2 Department of Civil and Environmental Engineering, Korea Advanced Institute of Science and Technology, 291 Daehak-ro, Yuseong-gu, Daejeon 34141, Korea

**Keywords:** seismic event detection, wireless sensor, IoT sensor, iBeacon, BLE, high fidelity sensing

## Abstract

The Internet of Things (IoT) has been implemented to provide solutions for certain event detection because of ease of installation, computing and communication capability, and cost-effectiveness. Seismic event detection, however, is still a challenge due to a lack of high-fidelity sensing and classification efficiency. This study proposes BLESeis, an IoT sensor for smart earthquake detection. BLESeis comprises three main parts: (1) high-fidelity vibration sensing using a MEMS accelerometer and digital filtering; (2) an embedded earthquake detection algorithm; (3) BLE (Bluetooth low energy) beacon for earthquake notification. For high-fidelity vibration sensing, a triggering algorithm and embedded finite impulse response (FIR) low-pass filter are developed. The acquired vibration is then classified by the earthquake detection algorithm developed to identify the earthquake signal from other vibration sources using time and frequency domain analysis. Upon detection of an earthquake, the BLE beacon broadcasts using the proposed data packet for efficient notification and visualization. The performance of the proposed system is evaluated through numerical simulations and a set of experiments using shaking table tests. The experiments show the feasibility of the low-cost earthquake detection and notification system.

## 1. Introduction

Earthquakes can threaten the lives of thousands of people in densely populated regions resulting in substantial financial loss. Earthquake early-warning (EEW) systems serve to detect the magnitude of an earthquake rapidly and alert many people to take protective actions such as covering and stopping trains [1,2]. Several studies have been conducted to enable real-time or early earthquake detection and warning systems [3,4,5,6,7,8,9,10,11,12,13,14,15,16]. For instance, Nakamura et al. [3] proposed an urgent earthquake detection and alarm system (UrEDAS) that uses not only maximum seismic motion but also an initial stage of the seismic motion to enable the early-stage detection. Although these studies proposed real-time and high-accuracy earthquake detection methods, numerous data would need to be collected from the seismic network with highly sensitive seismometers spread over the region. Therefore, current EEW systems based on traditional seismic and geodetic networks exist only in a few countries due to the high cost of installing and maintaining such systems.

Recently, the EEW system has been evolved with the rise in the Internet of Things (IoT) system driven by the convergence of technologies such as micro electro-mechanical systems (MEMS), wireless communication, and increased computing power [17,18,19,20,21,22,23]. At the early stages, the MEMS sensors were connected to PCs and comprised a seismic sensor network by publishing the data on the Internet. For example, Quake-Catcher Network (QCN) [17], Community Seismic Network (CSN) [18], and NetQuakes [19] were projects led by University of Stanford, the California Institute of Technology, and the United States Geological Survey (USGS) to detect earthquakes using low-cost MEMS sensors. Recently, EEW has been built for smartphones that have MEMS sensors, data processing, and communication capabilities. The iShake project [20] used an iPhone for measuring ground motion, intensity parameters and provided the first proof of principle that showed the ability of smartphones for earthquake motion capture. The MyShake [21], built on iShake, is a smartphone application that detects whether the movement of a phone is made by an earthquake or by other human activities and have the ability to recognize earthquake shaking from background noise by aggregating multiple recording of every smartphone. When a potential earthquake event is detected, preprocessed measurement data is sent rapidly to the centralized processing hub for accurate data processing and aggregation with other sensor data. However, as the current EEW is network-wise alert system using sensing clients and backend servers, residential or industrial areas that need customized alert, even without internet connectivity, may not benefit from the EEW.

In helping to resolve these issues, in this paper, a low-cost IoT sensor for earthquake detection, BLESeis was developed. BLESeis, built with BLE (Bluetooth low energy) technology, measures high fidelity vibration, processes seismic event data, and broadcasts results such as PGA (peak ground acceleration), and MMI-scale. Contributions of this paper are enumerated as follows: (1) BLESeis was designed with a MEMS accelerometer and a BLE integrated MCU (NRF52840BLE, Nordic) as a stand-alone seismic sensor that conducts data measurement, processing, and notification on board; (2) embedded software was developed to establish high-fidelity data measurement, seismic event detection; (3) custom BLE data packet was proposed for seismic event notification. The performance of the developed BLESeis was evaluated through a lab-scale test using a shaking table. The remainder of this paper is organized as follows. Section 2 proposes a developed hardware and software system for BLESeis, including data acquisition, and FIR filtering for low-pass filtering. Numerical and lab-scale experimental validation are presented in Section 3 and Section 4, respectively. The conclusion is given in Section 5.

## 2. Proposed BLESeis

**Figure 1:**
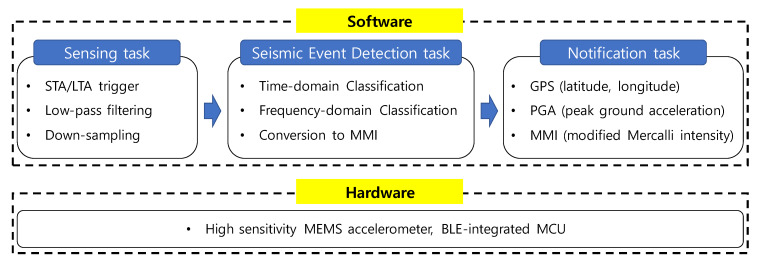
Hardware and software framework.

BLESeis is designed to deliver a reliable and fast customized alert to nearby Bluetooth devices. The hardware of the BLESeis consists of a high sensitivity MEMS accelerometer and BLE-integrated MCU. The software framework is divided into three main tasks: (1) sensing task, (2) seismic event detection task, and (3) notification task (see Figure 1).

### 2.1. Hardware Configuration

**Figure 2:**
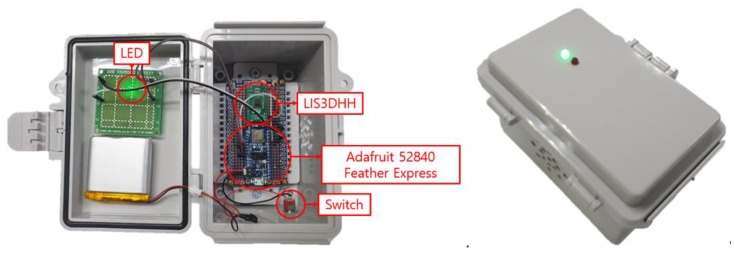
Developed BLESeis.

The IoT sensor platform used in the BLESeis is the Adafruit nRF52840 Feather Express (Adafruit industries, New York city, U.S.) that was developed based on nRF52840 (Nordic, 2019) with Arduino IDE support (see Figure 2); the nRF52840 is built with 32-bit ARM^®^ Cortex™-M4 CPU running at 64 MHz, allowing fast and efficient computation of complex functions requiring floating-point math for signal processing. nRf52840 provides extensive memory availability of 1MB and 256kB respectively in flash and RAM, enabling high-frequency sampling and computationally intense data processing required for seismic data acquisition and processing. Another salient feature of nRF52840 is the integration of Bluetooth 5 that enables low-power and long-range communication.

For high fidelity seismic data acquisition, the BLESeis uses a digital MEMS accelerometer, LIS3DHH, which features a high sampling of 1100 Hz and a low noise density of $45\;\mu g/\sqrt{Hz}$. Note that the noise density of LIS3DHH corresponds to the RMS level of 0.2 mg for 20 Hz bandwidth, which will be implemented in the sensing process. The detailed specification of the LIS3DHH is summarized in Table 1.

### 2.2. Sensing Task

**Figure 3:**
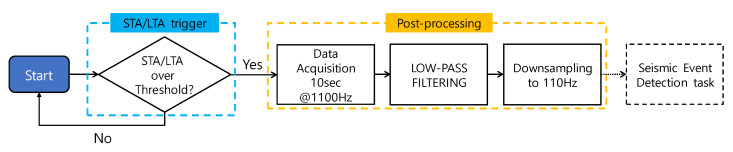
Flowchart of the sensing task.

The sensing task consists of STA (short-term average)/LTA (long-term average) trigger [24], low-pass filtering, and decimation (see Figure 3). When the STA/LTA trigger detects an event using the ratio of STA to LTA, the proposed BLESeis measures acceleration for 10 s. Afterward, measured data is low-pass filtered to remove unnecessary high-frequency noise and then downsampled to reduce the sampling rate by a factor of 10 for efficient memory use.

**Figure 4:**
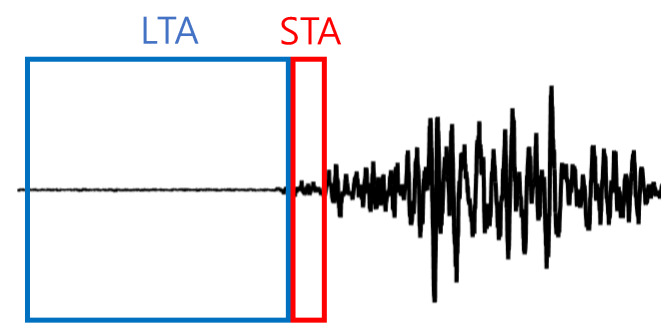
STA/LTA trigger.

The STA/LTA trigger is computationally efficient and the most widely used method for seismic vibration detection [25]. The STA measures the instantaneous amplitude of the newly input acceleration signal, while the LTA takes care of the current average of amplitude (see Figure 4). When the ratio of STA to LTA exceeds a threshold representing the event, the system is triggered.

**Figure 5:**
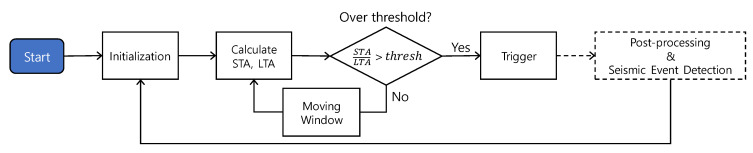
Implementation of the STA/LTA trigger.

The implementation of the STA/LTA algorithm for proposed BLESeis follows the flowchart, as shown in Figure 5. In the initialization step, acceleration is collected at a sampling frequency of 1100 Hz. STA and LTA are calculated based on predefined short-term and long-term window. Then, the ratio of STA to LTA is compared with the threshold; if the ratio of STA to LTA exceeds the preset threshold, the trigger is fired, otherwise moving window is applied to update LTA and STA. Note that in the implementation, each length of the predefined window for STA and LTA was 50 ms and 1000 ms, and the preset threshold was 1.3.

Once the trigger is activated, the acceleration is measured for 10 s at a sampling rate of 1100 Hz. In order to avoid aliasing, FIR (finite impulse response) low-pass filter [26] is applied, attributed to its stability and linear phase delay and ease of implementation.

**Figure 6:**
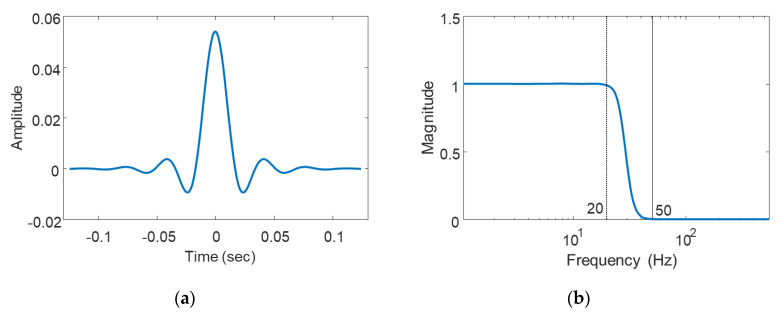
(**a**) Filter coefficient and (**b**) frequency response function.

Liu et al. have proposed transfer function for the low-pass filter expressed as

(1) $$H_{L}(f)=\frac{\beta_{L}{}^{2n}}{{\left(2\pi f\right)}^{2n}+\beta_{L}{}^{2n}}=\frac{1}{1+{\left(\frac{2\pi f}{\beta_{L}}\right)}^{2n}}$$

where f is the frequency, $\beta_{L}$ is the regularization factor, and n is the filter order. The regularization factor $\beta_{L}$ can be represented as

(2) $$\begin{array}{l} \beta_{L}=\sqrt[{n}]{\frac{\alpha_{L}}{1-\alpha_{L}}}2\pi f_{c}\\ \alpha_{L}=H_{L}(f_{c})=\frac{\beta_{L}{}^{2n}}{{\left(2\pi f_{c}\right)}^{2n}+\beta_{L}{}^{2n}} \end{array}$$

where $\alpha_{L}$ is the accuracy factor that indicates the magnitude of the transfer function at the cut-off frequency, $f_{c}$. Note that $f_{c}$ can be located at the edge of the pass band as $\alpha_{L}$ is close to 1. The filter coefficients for the implementation of the FIR filter can be approximated using inverse discrete Fourier transform as

(3) $$\begin{array}{l} C_{L}(p)=\frac{1}{f_{s}}\int_{-f_{s}/2}^{f_{s}/2}H_{L}(f)e^{-j2\pi pf/f_{s}}df\\ =\frac{2}{f_{s}}\int_{0}^{f_{s}/2}\frac{\beta^{2n}}{{\left(2\pi f\right)}^{2n}+\beta^{2n}}\cos(2\pi pf/f_{s})df\\ =\frac{2}{f_{s}}f_{c}\int_{0}^{1/2r_{T}}\frac{\beta^{2n}}{{\left(2\pi f_{c}\tilde{f}\right)}^{2n}+\beta^{2n}}\cos(2\pi pr_{c}\tilde{f})d\tilde{f} \end{array}$$

where $r_{c}=f_{c}/f_{s}$ and denotes the ratio of the cut-off frequency to the sampling frequency, and $\tilde{f}=f/f_{c}$ is the normalized frequency. In this study, the property of the low-pass filter designed in with the cut-off frequency (i.e., *f_c_*) at 20 Hz, $\alpha_{L}$ of 0.99, and the filter order *n* of 7. The cut-off frequency is determined to remove unnecessary noise above 20 Hz to improve the accuracy of the earthquake detection. The length of the filter is truncated to 275 samples (i.e., 250 ms) for efficient filter implementation. Figure 6 shows the 275 filter coefficients and transfer function, which have the pass band region up to 20 Hz and the transient band up to 50 Hz. The filtered acceleration is downsampled by a factor of 10, to make a sampling rate of 110 Hz.

### 2.3. Seismic Event Detection Task

**Figure 7:**
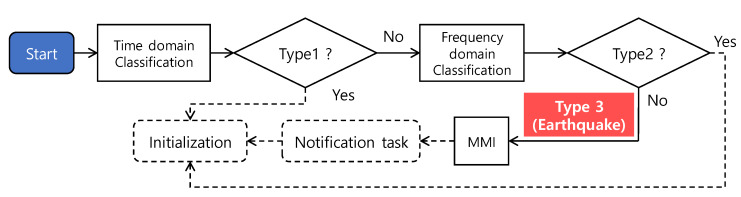
The flow of the seismic event detection task.

The seismic event detection task analyzes the acquired signal in the time and frequency domain to distinguish earthquake from type 1-stationary signal and type 2-non-stationary signal, including a few major frequency components (see Figure 7). Note that the proposed detection algorithm classifies signals into three types: type 1 (random vibration), type 2 (structural vibration), and type 3 (earthquake). The proposed detection algorithm first distinguishes the type 1 signal and potential earthquake in the time-domain analysis followed by frequency-domain analysis for classifying earthquakes from the nonstationary signal.

Let X(i),  i=1,…,1100 be a sample from a measured signal for 10 s at 110 Hz, which was downsampled from 1100 Hz (i.e., 1100 data points). Then, four windowed segments of the length of *N* without overlap were taken. Let $X_{k}\left(i\right),\;\;k=1,2,3,4$ be the segments of each windowed sample such that $X_{k}\left(i\right)=X\left(i+(k-1)N\right)$, where i=1,…,N and *N* is 275 samples, which is a quarter of the total data size. The type 1 signals such as random and harmonic oscillations can be classified using the stationarity index of the signal defined as

(4) $$\frac{\min(\sigma_{k})}{\max(\sigma_{k})}>\tau_{random}$$

where $\sigma_{k}$ is the standard deviation of each windowed signal $X_{k}$, and $\tau_{random}$ is the threshold for type 1 signal classification. In this study, we set $\tau_{random}$ as 0.75, considering measurement noise.

After the time-domain classification, the frequency-domain analysis was conducted to detect the earthquake signal. The PSD (power spectral density) of the measured signal $S_{xx}(i)\;\;(1\leq i\leq 512)$ was calculated with the number of Discrete Fourier transforms of 1024, such that the frequency spectral resolution has 0.01 Hz (i.e., 110 Hz/1024). Let $N_{s}$ be the number of elements of $S_{TH}$ defined as

(5) $$S_{TH}\;=\;\left\{v|v>E\left[S_{xx}\right]\;+\;\lambda \sigma_{S_{xx}},\;\forall_{v}\in S_{xx}\right\}$$

where λ is adjusting factor and σ is standard deviation of $S_{xx}$. Given the distribution of frequency components of an earthquake, the signal is classified as an earthquake if $N_{s}>\tau_{seismic}$, where $\tau_{seismic}$ is an adjustable threshold. In this study, λ of 0.5 and $\tau_{seismic}$ of 25 are used, respectively. The MMI was obtained using peak measured acceleration.

### 2.4. BLE for Seismic Event Notification

**Figure 8:**
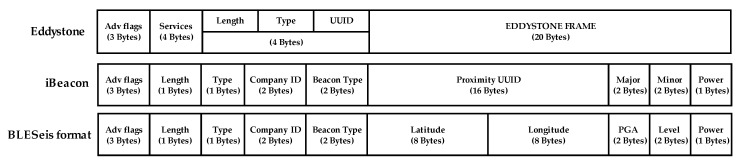
BLE packet format.

BLE is the term for Bluetooth 4.0+ and is characterized by low power consumption, and it has the ability to exchange data either in connection or advertising mode [27]. In advertising mode, BLE devices can broadcast certain information (e.g., temperature, battery status, etc.) without direct connection to neighboring devices. Advertising mode uses the generic access profile (GAP) layer to broadcast data, specially formatted advertising packets, in a one-to-many transfer. Each type of beacon uses a custom specification to partition up the advertising data according to the SIG (signal interest group) specifications. For instance, iBeacon and Eddystone are the commonly used data packet (31 Bytes) for the BLE beacon (see Figure 8). iBeacon, developed by Apple, is the first beacon protocol introducing the beacon to broadcast their identifier to nearby portable electronic devices. Eddystone was the protocol developed two years later by Google. Eddystone has more flexibility in data packets and compatibility with both Android and IOS. However, using and modifying data packets requires a more complex programming process compared to iBeacon.

This paper proposes a BLESeis protocol to more effectively advertise seismic information (i.e., location of the sensor, peak acceleration, MMI scale, and transmission power (TX power)) to surrounding IoT devices. The proposed protocol was created by modifying iBeacon’s data packets without affecting the standard. Note that iBeacon protocol was selected because of ease of modification for the intended use (i.e., UUID for GPS, Major for PGA, and Minor for level). The definition of the BLESeis data packets is given as:

- Company ID (2-Byte): Identifier of the manufacturer of a BLESeis (e.g., BS (0x4353))
- GPS coordinate: (16-Byte): GPS latitude (8-Byte) and longitude (8-Byte) of the BLESeis (e.g., Lat: 37.503640480778266, Long: 126.95702612400056 for Chung-Ang University, Seoul, Korea)
- PGA (2-Byte): Maximum measured acceleration in mg unit. (e.g., 34)
- Level (2-Byte): MMI scale of PGA multiplied by 10
- TX (1-Byte): TX power level, indicating the signal strength of the BLE device when transmitted

## 3. Numerical Validation

### 3.1. Numerical Setup

The numerical simulation was conducted to validate the performance of the STA/LTA detector and seismic event detection algorithm. The artificial earthquake, structural vibration, and random signal were prepared; the artificial earthquake was generated by Kanai–Tajimi [28,29,30,31,32] spectrum whose transfer function is expressed as:

(6) $$S(\omega )=S_{0}\frac{\left[1+\xi_{g}^{2}{\left(\omega /\omega_{g}\right)}^{2}\right]}{{\left[1-{\left(\omega /\omega_{g}\right)}^{2}\right]}^{2}+4\xi_{g}^{2}{\left(\omega /\omega_{g}\right)}^{2}}$$

where $S_{0}$ is the power of the random input signal $\xi_{g}^{}$, $\omega_{g}^{}$ are soil damping and soil frequency, respectively. In the numerical simulation, $\xi_{g}^{}$, $\omega_{g}^{}$ are set to 0.3 and 17 rad/s to generate an artificial earthquake in the specific ground condition. The type 2 vibration that is modeled as a combination of two sine wave, $v(t)=A_{1}\sin(2\pi f_{1}t)+A_{2}\sin(2\pi f_{2}t)$ which follows normal distribution as:

(7) $$\begin{array}{l} f_{1}\sim N(0.5,1)\\ f_{2}\sim N(2,1)\\ A_{1},A_{2}\sim N(0.5,1) \end{array}$$

Note that the Hanning window [33] is applied to smoothen the seismic and type 2 vibration. Type 1 vibration was modeled as a random signal having power spectral density of $S_{0}$ without the Hanning window.

### 3.2. STA/LTA Trigger

**Figure 9:**
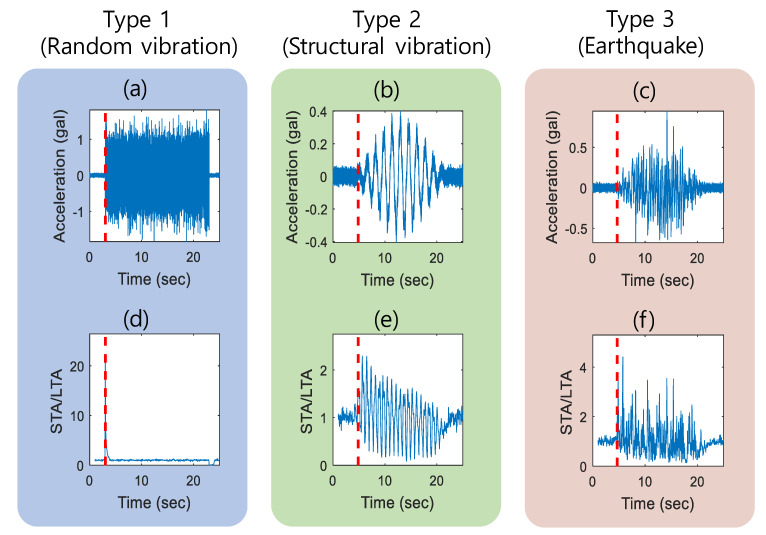
Examples of sampled acceleration and trigger: (**a**) random vibration; (**b**) structural vibration; (**c**) artificial earthquake; (**d–f**) STA/LTA ratio and detected triggering time (red).

With a total of 300 vibration datasets consisting of 100 datasets for each case (i.e., type 1, type 2, and type 3–earthquake), the detection of the trigger was validated as shown in Figure 9 with the red line that represents the time of trigger detected by the STA/LTA algorithm. STA/LTA ratio showed an abrupt change at the occurrence of the vibration, whereas the ratio is 1 for the vibration-free region. Figure 9a shows the time-domain acceleration of type 1 (e.g., random vibration), and Figure 9d shows the corresponding ratio of STA/LTA. Since the signal is stationary after a certain point, the STA/LTA ratio shows a peak point that is the time of the trigger. Figure 9b,c show type 2 (e.g., structural vibration) and type 3 signal (e.g., earthquake), respectively. Since both type 2 and type 3 signals are nonstationary, many peaks in the ratio of STA/LTA are shown in Figure 9e,f.

The STA/LTA algorithm successfully detected the change in vibration and showed a high precision and reliable detection capability by showing the time of trigger at 3000 ms for type 1, 4713 ± 76 ms for an earthquake, and 4994 ± 791 ms for type 2. The time of trigger can differ by types of vibration as type 2 and type 3 (earthquake) vibrations are smoothed by the Hann window that delayed the occurrence of the vibration, whereas type 1 vibration caused an abrupt change in the STA/LTA ratio. The STA/LTA ratio can be adjusted depending on the site condition; if there is no ambient source of vibration, STA/LTA is set to low to increase detection sensitivity; the site with ambient vibration source can have higher STA/LTA to make more robust detection lowering the false-positive rate.

### 3.3. Performance of the Proposed Seismic Event Detection

**Figure 10:**
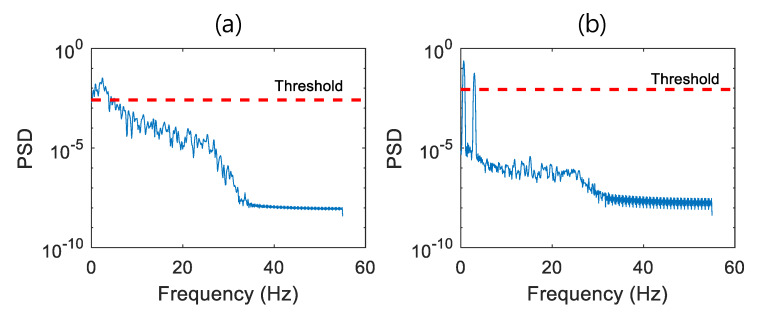
Power spectral density (PSD) of (**a**) the generated earthquake and (**b**) structural vibration.

A total of 300 datasets were evaluated using the proposed earthquake detection algorithm. The seismic event detector first classifies the stationary and nonstationary signal using Equation (4) with the $\tau_{random}$ of 0.75. In this time-domain analysis step, 100 type 1-stationary vibration datasets were all classified as type 1 vibration. A nonstationary signal that passed time-domain analysis was classified by frequency-domain analysis given in Equation (5). Figure 10 shows the PSD of generated seismic wave and type 2 with a pre-determined threshold of $E\left[S_{xx}\right]+0.5\sigma_{s_{x}{}_{x}}$. Using the number of PSD data points exceeding the threshold, the seismic wave and type 2 can be successfully classified. For example, the signal in Figure 10a is classified as seismic wave because the number of PSD data points exceeding the threshold was greater than 25, whereas the signal in Figure 10b had only two data points exceeding the threshold and was classified as type 2 signal. Out of 200 nonstationary signals consisting of the generated earthquake and type 2, all the vibration were successfully classified as seismic wave and type 2 signal, respectively, without a single misclassification.

## 4. Experimental Validation

The performance of developed BLESeis was evaluated experimentally through two types of tests: ambient vibration tests and shaking table tests. Ambient vibration tests were conducted to demonstrate the low-noise level and performance of the embedded filter of the sensor. The shaking table tests were conducted on a shaking table with a generated random, earthquake, and type 2 vibration in order to demonstrate the performance of the seismic event detection.

### 4.1. Ambient Vibration Test on BLESeis

The ambient vibration tests were conducted to measure the noise floor of developed BLESeis and compared with the amplitudes of the various magnitude of earthquakes measured at the distance of 10 km [34].

**Figure 11:**
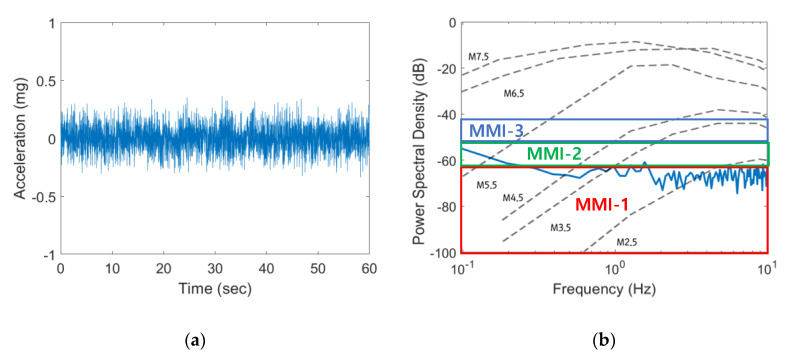
Noise floor of developed BLESeis: (**a**) time-domain noise; (**b**) frequency-domain noise.

Figure 11 shows that developed BLESeis has sensitivity to magnitude 3.5 (M3.5) and MMI-2 (PGA of 0.7 mg) or larger earthquakes in the frequency range of 1 to 10 Hz, which is the most critical frequency component of an earthquake that cause the most damage.

### 4.2. Shaking Table Test

To validate the seismic event detection efficiency of the developed BLESeis, three types of vibration were excited by the shaking table: (1) type 1-random, (2) type 2-vibration with different driving frequencies, and (3) type 3 – artificial earthquake. The three types of vibration followed the design made in the numerical validation. The peak magnitude of each vibration generated by the shaking table was adjusted to have an MMI scale of 5 and 7. A total of 60 tests were conducted to validate the performance of developed BLESeis and were compared with the reference accelerometer.

**Figure 12:**
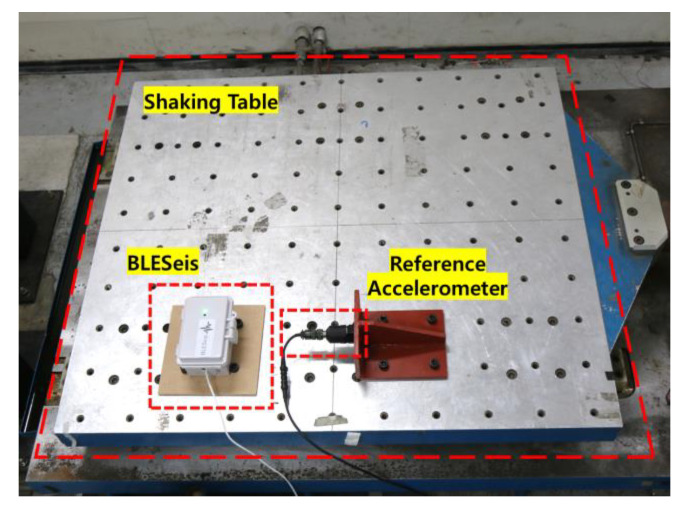
Experimental Setup.

Figure 12 shows the experimental setup with developed BLESeis and reference accelerometer fixed on the 1-dof shaking table. The sampling rate of reference accelerometer was set to 100 Hz for comparison with developed BLESeis. Each vibration was designed to have 10 s of delay before the main event occurred. Note that, due to the ambient vibration of the shaking table, vibrations with a small MMI scale were not considered in the experiments. In the experiment, the parameters related to triggering, STA/LTA,$\tau_{random}$, λ, $\tau_{seismic}$ were set 1.3, 0.75, 0.5, and 25, respectively.

**Figure 13:**
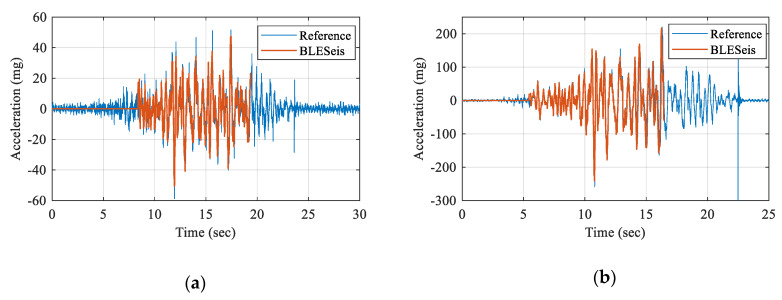
Comparison of BLESeis with reference acceleration: (**a**) artificial earthquake MMI-5; (**b**) type 2 vibration MMI-7.

Figure 13 shows the measured acceleration of BLESeis compared with the reference accelerometer. The measurement was triggered at the beginning of the occurrence of the vibration, and 10 s of data were acquired. The measured acceleration of BLESeis showed good agreement with reference acceleration, validating the performance.

The detection capability of the BLESeis was evaluated through a total of 60 experiments using different loadings, and the results are summarized in Table 2. It was shown that the proposed BLESeis successfully detected seismic events as well as classifying type 1 and type 2 signals.

**Figure 14:**
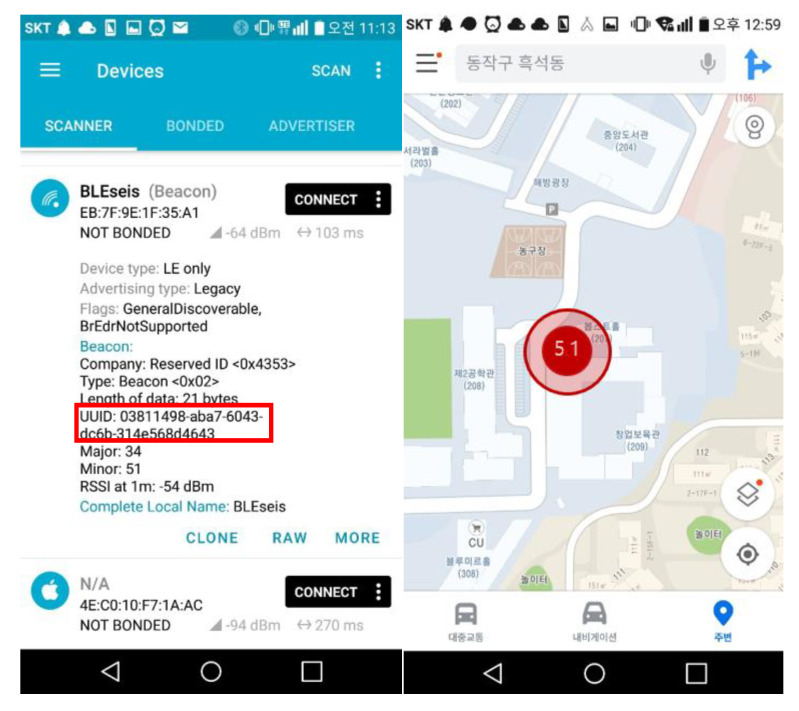
Visualization of BLESeis Packet.

The proposed beacon packet was captured and visualized through a smartphone (see Figure 14). The UUID highlighted by the red box indicates the location (i.e., latitude and longitude) of the BLESeis. The major and minor are the PGA in mg unit, and MMI-scale multiplied by 10, respectively. The BLESeis packet was visualized with its location and MMI-scale, which can be transmitted to any BLE devices nearby.

## 5. Conclusions

In this article, the BLESeis, which can detect earthquakes using low-cost MEMS sensors, is introduced. The hardware and software framework were designed carefully for the BLESeis to enable STA/LTA trigger, high-fidelity vibration sensing, and earthquake detection using time and frequency domain analysis. Moreover, the beacon packet is designed to efficiently broadcast the sensor’s location, PGA, and MMI-scale. The numerical simulation was conducted to validate the performance of the proposed detection algorithm, and parameters associated with triggering and detection are set accordingly. The experimental validation was carried out on a shaking table excited by random vibration, vibration with two major modes, and earthquake. The experimental results showed that the developed BLESeis detects the occurrence of a vibration and successfully classifies an earthquake with 100% accuracy. Moreover, the BLE beacon packets were shown to broadcast with defined information, including location, PGA, and MMI-scale that can be used for a customized alert system.

Note that, even though the proposed sensor showed remarkable performance, it has the following limitations: (1) the notification range is limited to 10 m with BLE, so that long-range communication is difficult. The proposed sensor used BLE for close-range notification with advertising mode, and it cannot transfer the notification to distant devices; (2) the parameters of the proposed algorithm, such as the ratio of STA/LTA, should be re-defined according to the properties of the ground or place where the sensors will be deployed. Numerical simulation considering specific ground properties might have to be conducted.

Future research will be conducted to develop a low-power sensor that can be used for long-term monitoring of a seismic event. Furthermore, long-range earthquake notification using ZigBee or LoRa will be researched to address the limitation of the notification range.

## Figures and Tables

**Table 1:** Specification of BLESeis.

Parameter	Value
Clock speed	32-bit ARM CortexM4 @ 64
RAM/FLASH	256kB/1MB
BLE distance	200 m +
Measurement range	±2.5 g
Noise density	45 μg/$\sqrt{Hz}$
Sensitivity	0.076mg/digit
Data output	16 bit
Sampling rate/Low-pass cut-off frequency	1100 Hz/200 Hz
Power consumption (mA)	14.3

**Table 2:** Event detection accuracy.

			Prediction				
			Type 1	Type 2	Type3: Earthquake	Total	%
**Actual**	Type 1	MMI-5	10	0	0	10	100
		MMI-7	10	0	0	10	
	Type 2	MMI-5	0	10	0	10	100
		MMI-7	0	10	0	10	
	Earthquake	MMI-5	0	0	10	10	100
		MMI-7	0	0	10	10	
	Total		20	20	20	60	100
